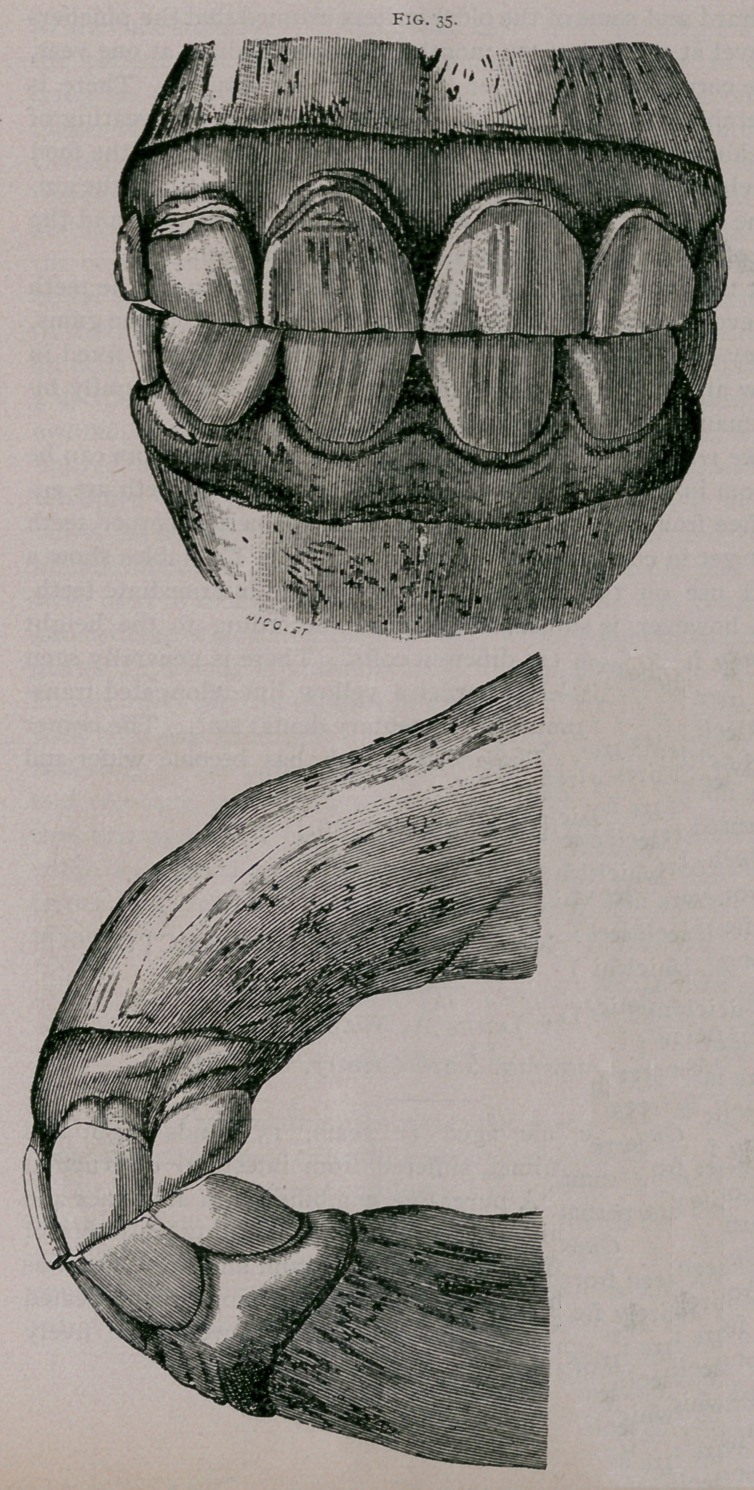# Age of the Horse, Ox, Dog, and Other Domesticated Animals

**Published:** 1890-08

**Authors:** R. S. Huidekoper

**Affiliations:** Veterinarian


### AGE OF THE HORSE, OX, DOG, AND OTHER DOMESTICATED ANIMALS.

BY R. S. HUIDEKOPER, M.D., VETERINARIAN.

[*Continued from page 397.*]

*Second Period.*—Leveling, progressive use and falling out of the incisors of first dentition.

*About one year.*—The corner teeth have protruded from the gums, but the inferior ones are not yet in contact with the superior teeth. The inferior pinchers are about leveled, before very much use on both their borders ; the incisive arch commences to

FIG. 32.

be a little depressed in the centre. The superior pinchers and intermediate teeth commence to wear at their posterior borders (Fig. 32).

FIG. 32.

FIG. 33.

FIG. 33.

*About sixteen months.*—The superior corner teeth are in apposition with the inferior, and have commenced to be leveled in both jaws; the crown of the tooth is entirely free from the gum. Often at this period the inferior pinchers are leveled, and sometimes the inferior intermediate teeth are also leveled. In the upper jaw the table of both teeth is entirely formed; a depression of the superior incisive arch is found (Fig. 33).

FIG. 34.

*About twenty months.*—The inferior corner teeth are leveled on their anterior border; the superior corners are somewhat worn. The inferior pinchers are completely worn, and the inferior intermediate teeth are often leveled; usually, the incisive arch becomes less convex (Fig. 34).

FIG, 34.

FIG. 35.

*About two years.*—The inferior dental arch is completely worn to the level at the pinchers and intermediate teeth. The superior arch is less worn ; the crown of the superior pincher teeth stands out from the gum. Pressure on the palatine arch, posterior to these teeth, shows a sensibility of the gum, and the permanent teeth can be felt under the soft tissues. The intermediate teeth are free from the gum ; the incisive arch becomes wider and is flattened in the centre (Fig. 35).

FIG. 35.

Girard and some of the older writers claimed that the pinchers were level at the end of ten months, the intermediate at one year, and the corner teeth at fifteen to twenty-four months. There is considerable variation and want of regularity in the wearing of the deciduous teeth, due to the amount and character of the food on which the foal or colt has been raised. The health of the animal, the amount of cement which fills the dental cup, and the nature of the food, all influence the result of friction.

At the end of this second period the free portions of the teeth have developed in length ; their necks become free from the gums, and they become brownish in color ; they are less solidly fixed in the jaw and may be broken off, or are pushed out naturally by the permanent teeth, which replace them.

*One year.*—(Fig. 32.) All of the deciduous incisors can be seen from in front ; the pinchers and intermediate teeth are entirely free from the gums. In profile the superior corner teeth are not yet in contact with the inferior teeth. The tables show a decided use on the posterior borders of the intermediate teeth, which, however, is subject to variation, according to the height of the posterior border in different colts. There is generally seen at this time in the anterior border a yellow line, elongated transversely, which represents the elementary dental star. The corner teeth are still virgin. The incisive arch has become wider and less rounded in the middle.

[TO BE CONTINUED.]

## Figures and Tables

**Fig. 32. f1:**
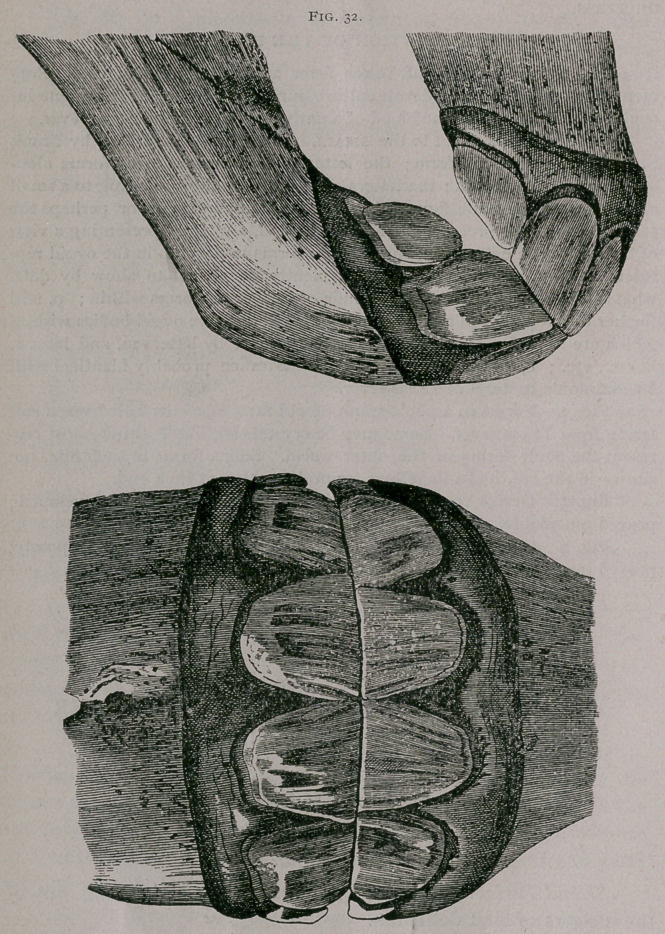


**Fig. 32. f2:**
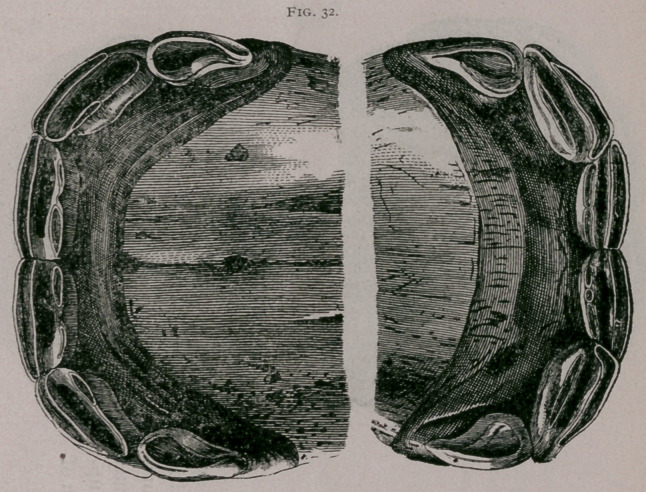


**Fig. 33. f3:**
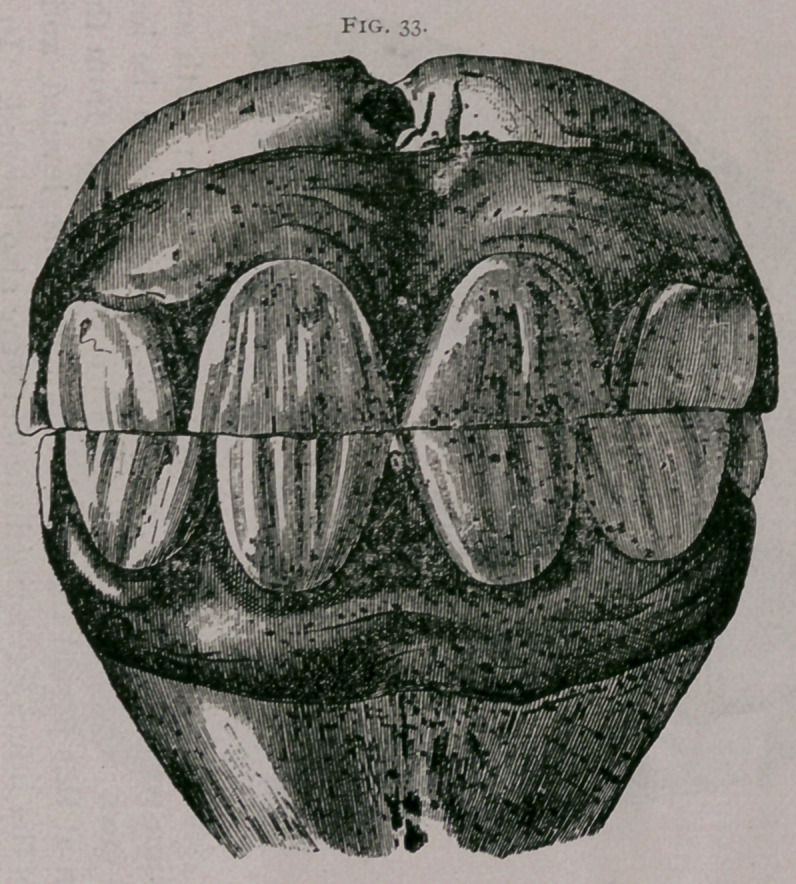


**Fig. 33. f4:**
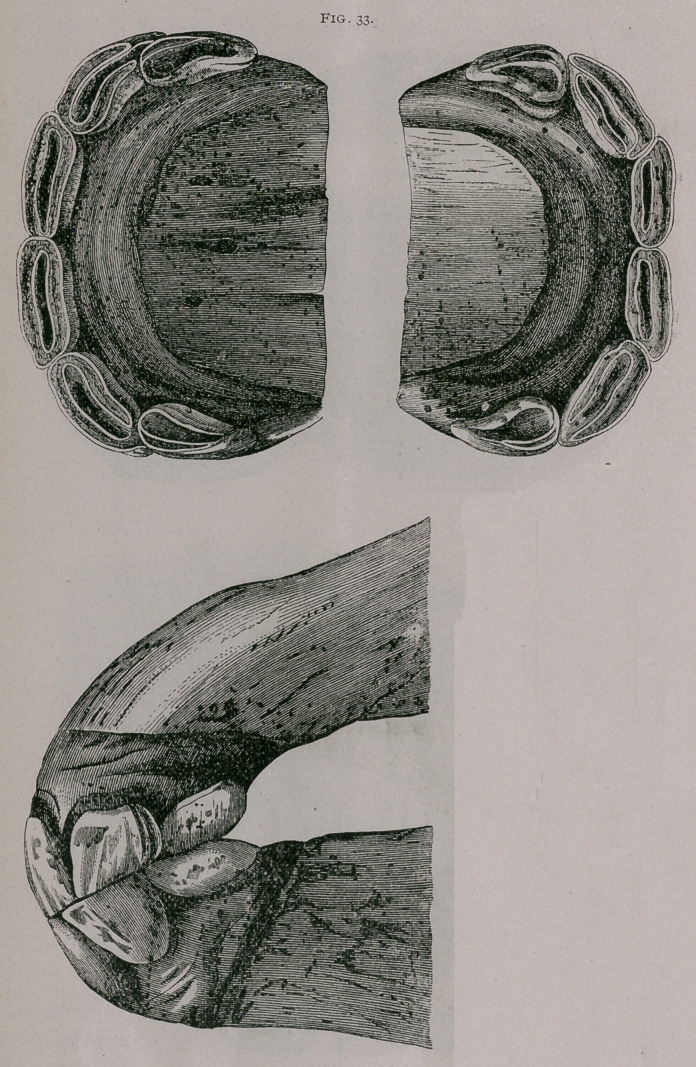


**Fig. 34. f5:**
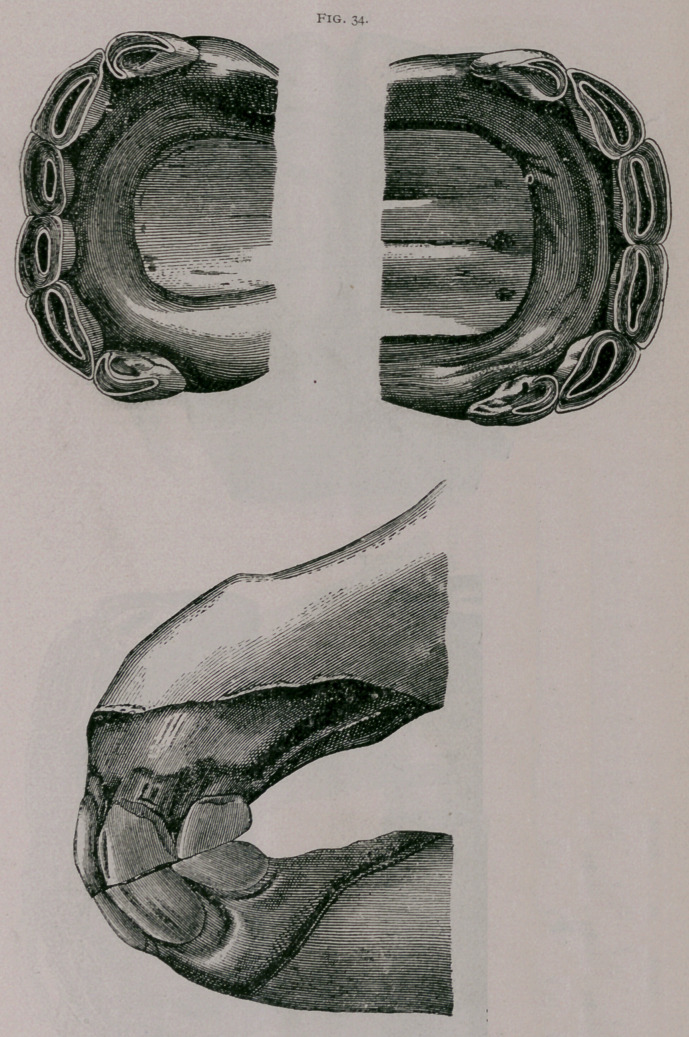


**Fig. 34. f6:**
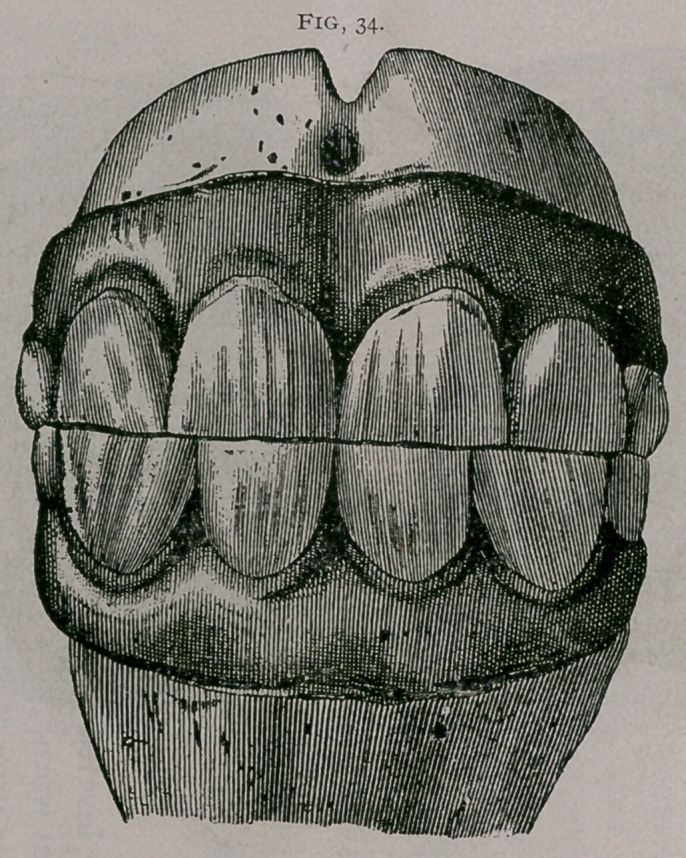


**Fig. 35. f7:**
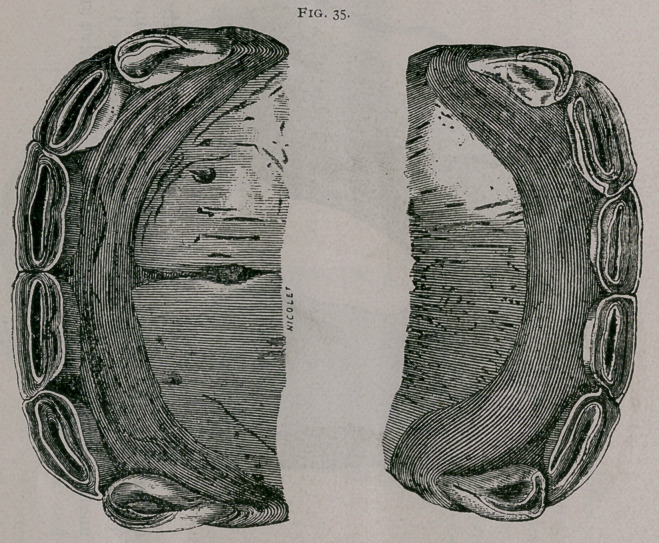


**Fig. 35. f8:**